# Temporal transcriptome and metabolome study revealed molecular mechanisms underlying rose responses to red spider mite infestation and predatory mite antagonism

**DOI:** 10.3389/fpls.2024.1436429

**Published:** 2024-08-14

**Authors:** Yanfei Cai, Ziming Shi, Peifei Zhao, Yingjie Yang, Yinshan Cui, Min Tian, Jihua Wang

**Affiliations:** ^1^ Flower Research Institute of Yunnan Academy of Agricultural Sciences, Kunming, Yunnan, China; ^2^ Yunnan Flower Technology Innovation Center, Kunming, Yunnan, China; ^3^ Yunnan Seed Laboratory, Kunming, Yunnan, China; ^4^ Yunnan Pulis Biotechnology Co. Ltd., Kunming, Yunnan, China

**Keywords:** *Tetranychus urticae*, *Neoseiulus californicus*, rose, transcriptome, metabolome, plant

## Abstract

**Introduction:**

Red spider mite (*Tetranychus urticae*) infestation (SMI) is a detrimental factor for roses grown indoors. Although predatory mite (*Neoseiulus californicus*) antagonism (PMA) is often utilized to alleviate SMI damage, little is known about the defensive response of greenhouse-grown roses to SMI and the molecular mechanism by which PMA protects roses.

**Methods:**

To determine the transcriptome and metabolome responses of roses to SMI and PMA, the leaves of a rose cultivar (“Fairy Zixia/Nightingale”) were infested with *T. urticae*, followed by the introduction of predator mite. Leaf samples were collected at various time points and subjected to transcriptome and metabolome analyses.

**Results:**

We found that 24 h of SMI exerted the most changes in the expression of defense-related genes and metabolites in rose leaves. KEGG pathway analysis of differentially expressed genes (DEGs) and metabolites revealed that rose responses to SMI and PMA were primarily enriched in pathways such as sesquiterpenoid and triterpenoid biosynthesis, benzoxazinoid biosynthesis, stilbenoid, diarylheptanoid and gingerol biosynthesis, phytosterol biosynthesis, MAPK signaling pathway, phenylpropanoid biosynthesis, and other pathways associated with resistance to biotic stress. Rose reacted to SMI and PMA by increasing the expression of structural genes and metabolite levels in phytosterol biosynthesis, mevalonate (MVA) pathway, benzoxazinoid biosynthesis, and stilbenoid biosynthesis. In addition, PMA caused a progressive recover from SMI, allowing rose to revert to its normal growth state. PMA restored the expression of 190 essential genes damaged by SMI in rose leaves, including transcription factors DRE1C, BH035, MYB14, EF110, WRKY24, NAC71, and MY108. However, after 144 h of PMA treatment, rose responsiveness to stimulation was diminished, and after 192 h, the metabolic levels of organic acids and lipids were recovered in large measure.

**Conclusion:**

In conclusion, our results offered insights on how roses coordinate their transcriptome and metabolome to react to SMI and PMA, therefore shedding light on how roses, *T. urticae*, and *N. californicus* interact.

## 1 Introduction

Roses (*Rosa chinensis*) are among the most significant horticultural plants, cultivated in various regions for their diverse flower shapes and vibrant colors. Furthermore, roses find extensive applications in the cosmetics and food industries, with considerable economic value (Cui et al., 2022). However, during intensive planting, roses are exposed to a variety of biotic and abiotic stresses, resulting in reduced yield. One of the biotic stresses affecting plants is insect feeding. The two-spotted spider mite (*Tetranychus urticae*) is regarded as a major pest that affect roses worldwide (Kang et al., 2007; Salman and Erbaş, 2014; Díaz-Arias et al., 2019). Despite current control methods, this insect continues to inflict enormous losses on the greenhouse rose industry, as spider mite thrive in warm and dry conditions (Field and Hoy, 1986), which are prevalent in greenhouse environments. Moreover, the excessive use of insecticides to manage *T. urticae* (Khajehali et al., 2011; Tirello et al., 2012) makes it impossible to disregard the negative effects of pesticide usage, such as environmental contamination, risks to human health, pest resistance, and secondary pest outbreaks (Taiwo, 2019; Leong et al., 2020; Yigit and Velioglu, 2020). The biological control of *T. urticae* with its natural foe, the predator mite *Neoseiulus californicus*, is viable, cost-effective, and ecologically benign.

The feeding methods of insects are generally divided into two types: piercing-sucking and chewing (Xue et al., 2022). Red spiders are piercing-sucking insects. They insert needle mouthparts into plant cells to feed, causing physiological changes in the plants. Plants have evolved specialized resistance mechanisms that enable them to detect exact environmental changes and adapt to unfavorable stress circumstances (Rejeb et al., 2014). Certain mechanisms, known as direct defenses, include the synthesis of antifeedant or toxin-inhibiting chemicals (Chen, 2008; Wu and Baldwin, 2010; Chuang et al., 2014). Other indirect defenses entail the release of herbivore-induced plant volatiles that attract the natural predators of the attacking herbivores (Howe and Jander, 2008; Turlings and Erb, 2018; Turlings et al., 1990). The buildup of phytohormones, including jasmonic acid (JA) (Rehrig et al., 2014), abscisic acid (ABA) (Vos et al., 2013), and salicylic acid (SA) (Schweiger et al., 2014), regulates plant resistance against insects. Plant-feeding insects often trigger JA-mediated signaling, which directly or indirectly activates both local and systemic defenses of plants (Kaur et al., 2010; Stam et al., 2014; Bozorov et al., 2017). In tobacco, SA and JA signals have been found to be activated in response to red spider infestation, triggering defense in both flowers and leaves (Munawar et al., 2023).

Insect feeding can induce an increase in the synthesis of lignin, cellulose, callose, alkaloids, phenolics, and silicon in plant leaves (Saunders et al., 1992; Moura et al., 2010; Nedukha, 2015; Kesten et al., 2017; Alhousari and Greger, 2018; Liu et al., 2018). These compounds bolster the toughness of leaves, forming a strong physical barrier that makes chewing by herbivores more difficult (Cesarino, 2019; Li et al., 2019). Moreover, plants may withstand insect through defense chemicals, which are insecticidal, antifeedant, antibacterial, and allelopathic. Such chemicals include benzoxazines, sesquiterpenes, flavonoids, isoflavonoids, phenylpropanes and stilbenes (Liu et al., 2006; Treutter, 2006; Guo et al., 2019; Habash et al., 2020; Valletta et al., 2021; Luo et al., 2022). Methyl ketones and zingiberene in tomato leaves have been found to display insect resistance by decreasing *T. urticae* fecundity rate and increasing nymph mortality (Antonious and Snyder, 2015; De Oliveira et al., 2018). Similarly, the buildup of other allochemicals, such as flavonoids in citrus, phenolic compounds in chrysanthemum, or terpenoids in cucumber and citrus, has also been linked to *T. urticae* resistance (Kielkiewicz and Van de Vrie, 1990; Balkema-Boomstra et al., 2003; Agut et al., 2014). This implies that secondary metabolites in plant leaves might provide plants with great insect resistance. Besides, phytosterols are also essential components of cell membranes and lipid rafts, essential for membrane integrity (Dufourc, 2008; Kumar et al., 2015; Zhang et al., 2020). To maintain membrane integrity and biological activities of lipid rafts, the relative sterol concentration is regulated in plants to cope with stresses. After inoculation with pathogens, plants have been demonstrated to strengthen the integrity of cell membranes by raising the stigmasterol/β-sitosterol ratio (Griebel and Zeier, 2010; Valitova et al., 2016; Cabianca et al., 2021). Studies have demonstrated that phytosterol-related genes, such as *SQS*, *SMT2*, and *C22-sterol desaturase*, are implicated in the immunological response to bacterial infections (Navarro Gallón et al., 2017; Zhang et al., 2020). However, little is known about the function phytosterols play in biotic and abiotic stress responses in plants.

Currently no thorough investigation has been conducted on the biochemical mechanism behind the impacts of spider mite infestation (SMI) and predatory mite antagonism (PMA) on rose. Our study addressed this gap by exploring the transcriptomic and metabolic responses of roses to SMI and PMA in a greenhouse setting. This research has significant implications for rose production, providing insights into the biochemical responses of roses to both pests and their natural predators. By examining the effects of *T. urticae* on roses from transcriptomic and metabolic perspectives, our study contributes valuable knowledge to the understanding of these alterations and offers potential strategies for improving rose cultivation and pest management.

## 2 Materials and methods

### 2.1 Plant materials

Roses used in this study were grown in the International Flower Technology Innovation Center (Kunming, China) in August and September in 2022. Cuttings (about 10-15 cm) of 1-year-old healthy roses of the “Fairy Zixia/Nightingale” cultivar were transferred to pots filled with coco coir. Afterwards, they were placed in a greenhouse free of red spiders for one month with water-soluble fertilizers applied once a week and normal watering. One month later, the cuttings grew into complete plants and were divided into three rows for subsequent experiments. The first and second rows were 35 pots each and used for infestation by *T. urticae*. The greenhouse settings were as follows: During the daytime, the temperature was 25°C, the humidity was 65%, and the light intensity was 70,000 lux. At night, the temperature was 16°C, and the humidity was below 85%.

### 2.2 *T. Urticae* infestation and *N. Californicus* antagonism

Two-spotted spider mites were used as herbivores. They were obtained by collecting leaves from another greenhouse of the innovation center. Using magnifying lens, rose leaves with spider mites were selected and carefully cut at the petiole with scissors. The obtained infested rose leaves were placed carefully to cover the leaves of the first two rows of the rose cuttings, leaving the third row as controls. After two hours of covering (8 -10 am), the cut leaves were removed. Spider mites that had successfully infested were counted by magnifying lens. *N. Californicus* was introduced as predators of *T. Urticae* and provided by Kunming Lixing Biotechnology Co., Ltd. After eight days (192 h) of spider mite infestation, predator mites were released on one row of the *T. urticae*-infested rose plants. Therefore, three rows of roses with different treatments were prepared for experiments: control (10 pots), SMI (35 pots), and PMA (35 pots).

### 2.3 Sample collection

Rose leaves of the *T. urticae*-infested (Tur) and control (CK) groups were collected at 24 h (Tur24, CK24), 96 h (Tur96, CK96), 144 h (Tur144, CK144), and 192 h (Tur192, CK196). Predator mites were released at 192 h of SMI, and rose leaves were collected at 144 h (Nca144) and 192 h (Nca192) of *N. Californicus* antagonism. All samples were immediately frozen in liquid nitrogen and stored at −80°C for subsequent experiments. All experiments were performed in triplicate.

### 2.4 Total RNA isolation and transcriptome sequencing

Plant total RNA was isolated using an RNAprep Pure Plant Plus Kit (Tiangen, China) following manufacturer’s instructions. The concentration and quality of RNA were determined using Agilent 5400 (Agilent, CA, USA) and 1% agarose gel electrophoresis. Then, mRNA was obtained with oligo beads and reverse transcribed to cDNA. The library was constructed using the NEBNext^®^ Ultra™ RNA Library Prep Kit for Illumina^®^ (NEB, USA) and quality determined by Qubit dsDNA HS Assay Kit. Qualified transcriptome libraries were paired-end (150 bp) sequenced on the Illumina NovaSeq6000 sequencing platform (Illumina, USA).

### 2.5 Metabolite extraction and quantification

Frozen plant samples were ground into powder at 30 Hz for 1 min. Fifty milligrams of powder were dissolved in 1 mL methanol/water/formic acid (15:4:1, V/V/V). Ten microliters of internal standard mixed solution (100 ng/mL) was added to each extract as internal standards (IS) for quantification. The mixture was vortexed for 10 min and then centrifuged for 5 min (12,000 r/min at 4°C). The obtained supernatant was transferred to a microtube, evaporated to dryness, dissolved in 100 mL 80% methanol (V/V), and filtered through a 0.22 mm membrane filter for further LC−MS/MS analysis.

The sample extracts were analyzed using a UPLC−ESI−MS/MS system. Phytohormones were analyzed using scheduled multiple reaction monitoring (MRM). Mass spectrometer parameters, including the declustering potentials (DP) and collision energies (CE) for individual MRM transitions, were performed with further DP and CE optimization. A specific set of MRM transitions was monitored for each period according to the metabolites eluted within the period.

### 2.6 Bioinformatics analysis

Clean reads of transcriptome sequencing were aligned to the reference genome RchiOBHm-V2 (https://www.ncbi.nlm.nih.gov/datasets/genome/GCF_002994745.2/) using HISAT2 (version 2.0.4). The counts of each gene was obtained using HTSeq-count program. The fragments per kilobase million (FPKM) value was calculated for gene expression levels. Statistical analysis of the data were performed using R (v.4.0.2). Differentially expressed genes (DEGs) were identified by DESeq2 (version 1.10.1), and enrichment analysis was conducted using clusterprofile. Genes with padj < 0.05 and absolute value of log2(FoldChange) ≥1 were identified as DEGs.

Metabolite abundances were quantified using the peak areas. Data obtained from metabolite profiling were analyzed by MetaboAnalystR. Principal component analysis (PCA) was performed and partial least squares-discriminant analysis (PLS-DA) model was built to obtain importance in project (VIP) values. Metabolites were defined as significantly differential metabolites (DIMs) when VIP ≥ 1 and fold change ≥ 2 (up-regulated) or ≤ 0.5 (down-regulated). Hypergeometric test was applied to identify the significant Kyoto Encyclopedia of Genes and Genomes (KEGG) pathways with a false discovery rate (FDR) < 0.05. Gene set enrichment analysis (GSEA) was conducted using the R package clusterProfiler (v. 4.10.0).

### 2.7 Quantitative real-time polymerase chain reaction analysis

Quantitative real time polymerase chain reaction (qRT-PCR) was used to determine the amount mRNA of key genes in samples. SYBR Green I SuperMix (Mei5 Biotech, Beijing, China) was used for qRT-PCR reaction. All primers used in this study are listed in **Supplementary Table 1**. The RT-qPCR was run with the following cycling conditions: 10 min of predenaturation at 95°C, then 95°C for 10 s, and 60°C for 30 s (40 cycles). The 18S gene was used as an internal control to calculate relative gene expression based on 2^−ΔΔCt^ method. All procedures were performed on three independent biological and technical repeats.

## 3 Results

### 3.1 Effects of SMI and PMA at various time points on rose transcriptome

**Figure 1:**
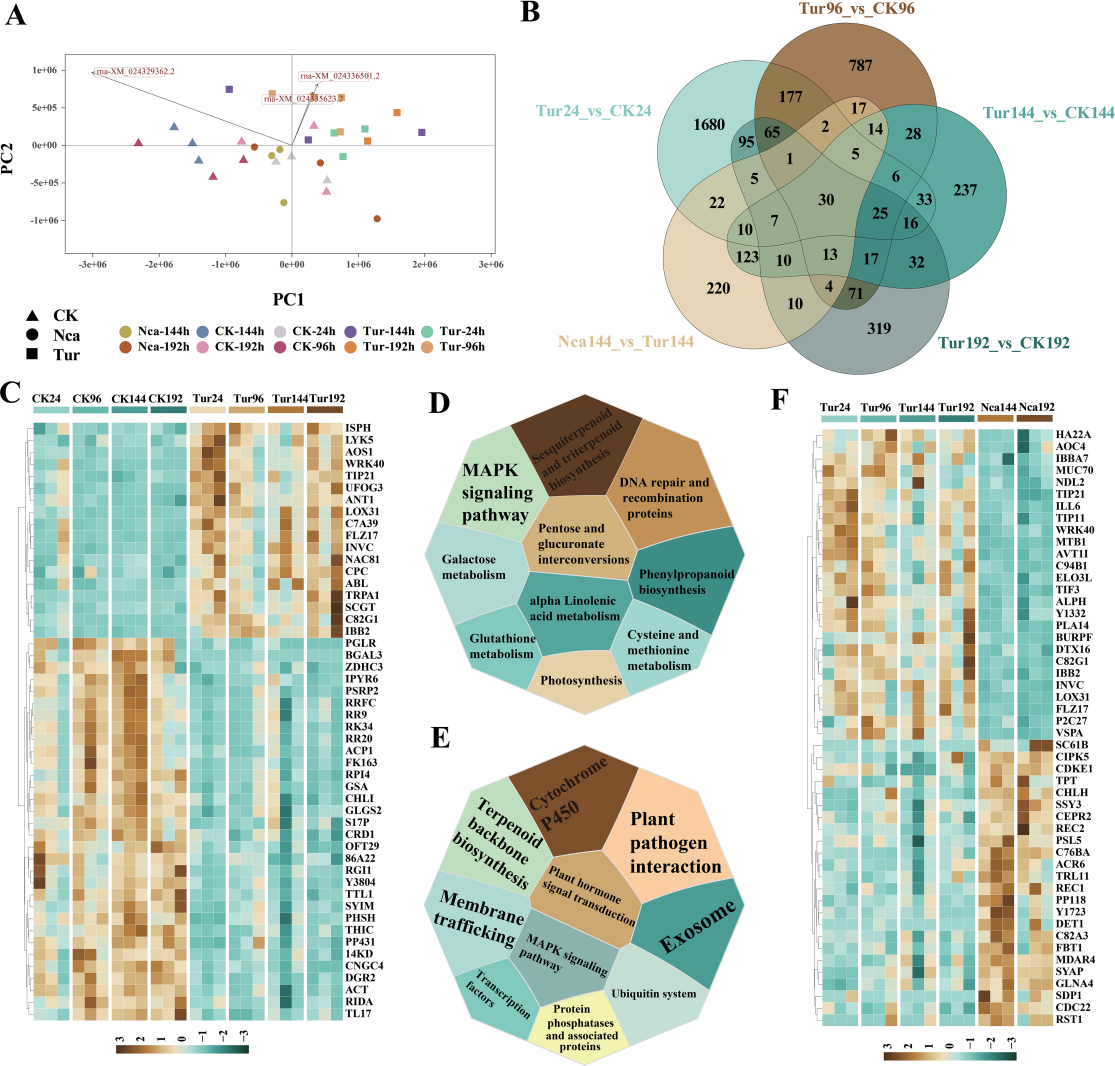
Transcriptome overview of the effects of spider mites infestation (SMI) and predatory mite antagonism (PMA) on rose leaves. **(A)** Principal Component Analysis (PCA) of the effects of SMI and PMA on rose leaves, where different colors represent different groups, and different shapes represent different treatments; triangles represent the control group, squares represent SMI treatment, and circles represent PMA treatment. **(B)** Venn diagram of differentially expressed genes (DEGs) between groups. **(C)** GSEA of genes in rose leaves after SMI treatment. This figure shows the 10 pathways with the highest NES value. **(D)** The most significantly changed 50 DEGs in rose leaves after SMI treatment. **(E)** GSEA of genes in rose leaves after PMA treatment. This figure shows the 10 pathways with the highest NES value. **(F)** The 50 DEGs with the most significant changes compared with SMI in rose leaves after PMA treatment.

To assess global transcriptomic shifts in response to SMI, control samples of CK24, CK96, CK144 and CK192 and corresponding time points by SMI (Tur24, Tur 96, Tur 144 and Tur 192) were analyzed, as well as PMA rose leaves at 144 h (Nca144), and 192 h (Nca192). All 60 samples were sequenced to yield a total of 201.79 G of raw data and a total of 197.09 G of filtered clean data (**Supplementary Table 2**). The clean reads were aligned to the reference genome, and the expression level of each gene was calculated. A total of 69,459 transcripts were detected in all samples, with further screening revealing 26,367 transcripts to be effectively expressed. PCA was used to unveil the overall difference in gene expression levels (**Figure 1A**). PC1 distinguished between the control samples and SMI samples, whereas PC2 distinguished between the SMI sample and PMA samples (**Figure 1A**). Both SMI and PMA substantially triggered gene expression alterations in rose leaf samples. We then identified 2179 DEGs (1192 up-regulated and 987 down-regulated), 1261 (742 up-regulated and 519 down-regulated), 605 (238 up-regulated and 367 down-regulated), and 720 (545 up-regulated and 174 down-regulated) in Tur24, Tur96, Tur144 and Tur192, respectively, compared with their corresponding control groups. Similarly, 492 DEGs (354 up-regulated and 138 down-regulated) and 389 DEGs (148 up-regulated and 241 down-regulated) were discovered in Nca144 (versus Tur192) and Nca192 (versus Tur192), respectively. A Venn diagram analysis found 1680, 787, 237, and 319 distinct DEGs in Tur24, Tur96, Tur144, and Tur192 compared with their corresponding control groups, indicating the specificity of rose transcriptome alterations at various time points following SMI (**Figure 1B**).

GSEA was then performed on all DEGs at each time point. DEGs shared by Tur24, Tur96, Tur144, and Tur192 were mostly engaged in photosynthesis, phenylpropanoid biosynthesis, cysteine and methionine metabolism, galactose metabolism, sesquiterpenoid and triterpenoid biosynthesis, MAPK signaling pathway, and galactose metabolism (**Figure 1C**). Genes such as *WRK40*, *LOX31*, *NAC81*, and *AOS1* were co-upregulated while *RR9*, *RR20*, *RRFC*, and *RK34* were co-downregulated (**Figure 1D**). This demonstrated a prolonged downregulation of genes involved in translation in rose leaves after SMI. The common DEGs between Nca144 and Nca192 were primarily involved in the pathways of plant pathogen interaction, exosome, Cytochrome P450 and terpenoid backbone biosynthesis (**Figure 1E**), including the common up-regulation of DEGs like *CIPK5*, *CDKE1*, *CEPR2*, *REC1*, *WRK40*, and *AOC4*, as well as co-downregulated genes like *LOX31* and *AVT1I* (**Figure 1F**). This demonstrated that PMA restored the cellular activity of rose leaves. To elucidate the molecular mechanism of biological control of SMI by PMA at various time points, we analyzed the gene expression level of PMA at rose leaves. GSEA revealed that 110 of the genes with restored expression from SMI in the Nca144 group were mostly involved in plant response to external stimuli, including response to acid chemical, response to biotic stimulus, response to external biotic stimulus, and response to wounding pathways (**Supplementary Figure 1**). These results showed the PMA indirectly recovered SMI rose gene expression.

### 3.2 Alterations in secondary metabolite synthesis and structural genes in response to SMI and PMA

**Figure 2:**
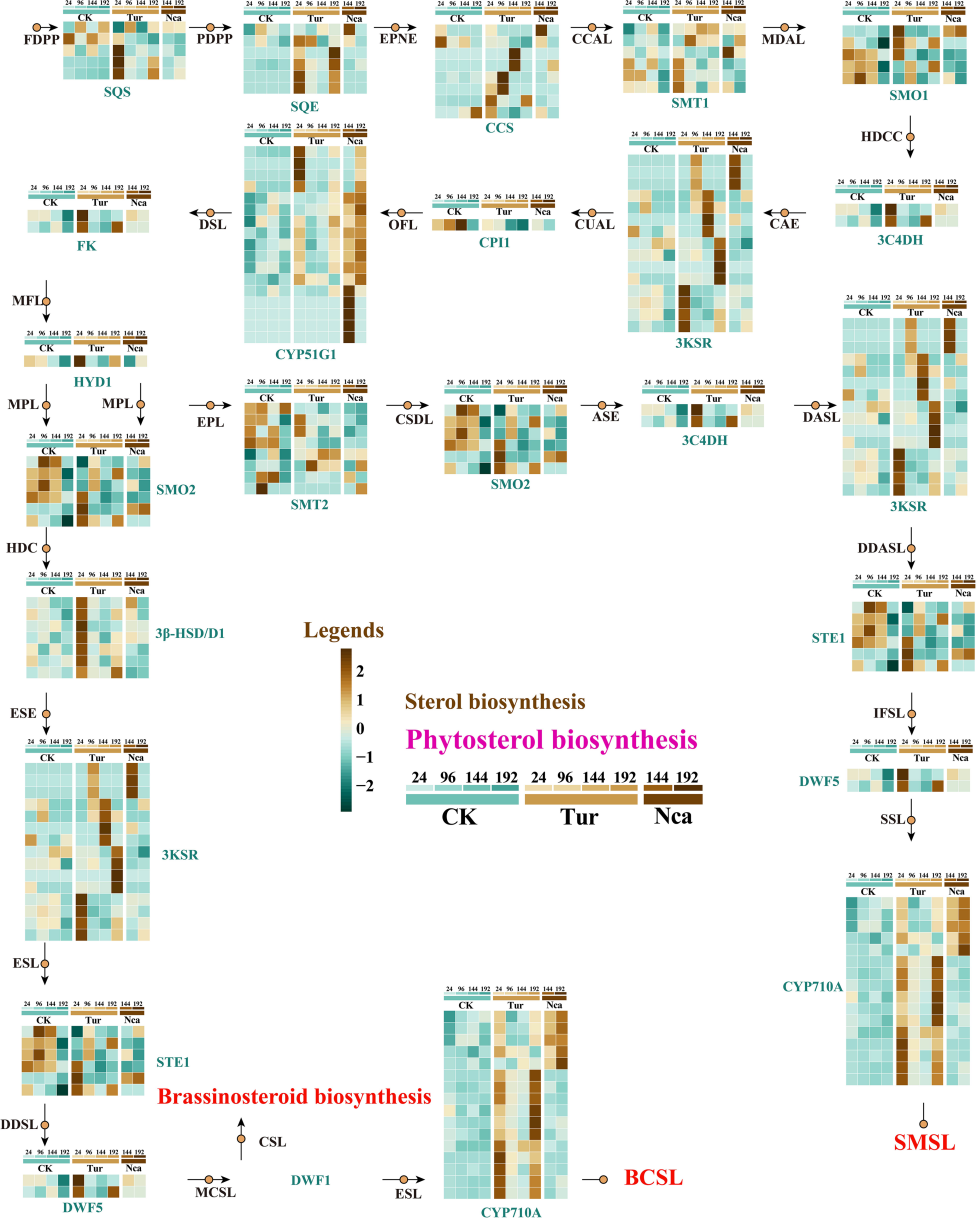
Reconstruction of the phytosterol biosynthesis pathway in rose leaves and expression changes of structural genes in response to SMI and PMA. Metabolites and structural genes are presented in the figure. The metabolites are abbreviated, and the meanings are as follows: FDPP, Farnesyl diphosphate; PDPP, Presqualene diphosphate; EPNE, (S)-2,3-Epoxysqualene; CCAL, Cycloartenol; MDAL, 24-Methylidenecycloartanol; HDCC, 5alpha-ergost-24(24(1))-en-4alpha-carboxylate; CAE, Cycloeucalenone; CUAL, Cycloeucalenol; OFL, Obtusifoliol; DSL, Delta8,14-Sterol; MFL, 4alpha-Methylfecosterol; HDC, 3beta-Hydroxyergosta-7,24(24(1))-dien-4alpha-carboxylate; ESE, Episterone; ESL, Episterol; DDSL, 5-Dehydroepisterol; MCSL, 24-Methylenecholesterol; CSL, Campesterol; EPL, Ethylidenelophenol; CSDL, 4alpha-Carboxy-stigmasta-7,24(24(1))-dien-3beta-ol; ASE, Avenastenone; DASL, Delta7-Avenasterol; DDASL, 5-Dehydroavenasterol; IFSL, Isofucosterol; SSL, Sitosterol; SMSL, Stigmasterol. In addition, the structural genes are as follows: *CYP51G1* (Sterol 14-demethylase), *FK* (Delta(14)-sterol reductase), HYD1 (3-beta-hydroxysteroid-Delta(8),Delta(7)-isomerase), *SMO2* (Methylsterol monooxygenase 2), *3β- HSD/D1*, *STE1* (Delta(7)-sterol-C5(6)-desaturase 1), *SMT2* (Cycloartenol-C-24-methyltransferase 2), *DWF5* (7-dehydrocholesterol reductase), *CYP710A* (Cytochrome P450 710A1).

We speculate that rose responds to the physical stimulation of red spider by regulating genes for the synthesis of secondary metabolites. As important components of cell membranes and lipid rafts, phytosterols are directly associated with membrane stability (Kumar et al., 2015; Moreau, 2019). To explore rose response to SMI and PMA, we assessed the metabolites and structural gene expression of the phytosterol pathway in rose. Stigmasterol levels were stable with 24 h of SMI, rose considerably at 96 h, and peaked at 144 h (**Supplementary Figure 2**). Then, we reconstructed the phytosterol biosynthetic pathway in rose, in which alterations in 17 structural genes in the pathway at various time periods of SMI and PMA treatment were demonstrated (**Figure 2**). Involved in the pathway were genes such as *SQS* (squalene synthase), *SQE* (squalene epoxy), *CCS* (Cycloartenol synthase), 4)-sterol reductase), *HYD1* (3-beta-hydroxysteroid-Delta(8), Delta(7)-isomerase), *SMO2* (Methylsterol monooxygenase 2), *3-HSD/D1*, and *STE1* (**Figure 2**). We found that with 24 h of SMI (Tur24), 3 *SQS* genes, 5 *SQE* genes, 2 *CCS* genes, 1 *HYD1* gene, 3 *SMO1* genes, 3 *SMT1* genes, 2 *3C4DH* genes, 4 *3KSR* genes, 3 *SMO1* genes, 3 *SMO2* genes, 3 *STE1* genes, 7 *3- HSD/D1* genes, 2 *DWF5* genes, and 14 *CYP710A* genes were significantly up-regulated. We speculated that the up-regulated expression of these structural genes in the Tur24 group may directly result in the up-regulation of stigmasterol in the Tur96 group. Similarly, 1 *SQE*, 1 *CCS*, 1 *SMT1*, 3 *3KSR*, 5 *CYP51G1*, 1 *SMO2*, and 5 *CYP710A* genes were significantly up-regulated at 144 h of PMA, which may be associated with the elevation of stigmasterol in Nca144 and Nca192 groups (**Figure 2**). In addition, 2 *SQS*, 4 *SQE*, 4 *CCS*, 1 *3C4DH*, 11 *3KSR*, 1 *HYD1*, 1 *DWF5*, and 12 *CYP710A* genes were up-regulated by 96, 144, and 192 h of PMI treatment (**Figure 2**). In response to adverse stress, phytosterols play essential roles as membrane components or signaling molecules. After SMI and PMA exposure, roses exhibited considerable changes in phytosterol concentration and synthetic gene expression, which may indicate their involvement in the biotic stress response of roses.

**Figure 3:**
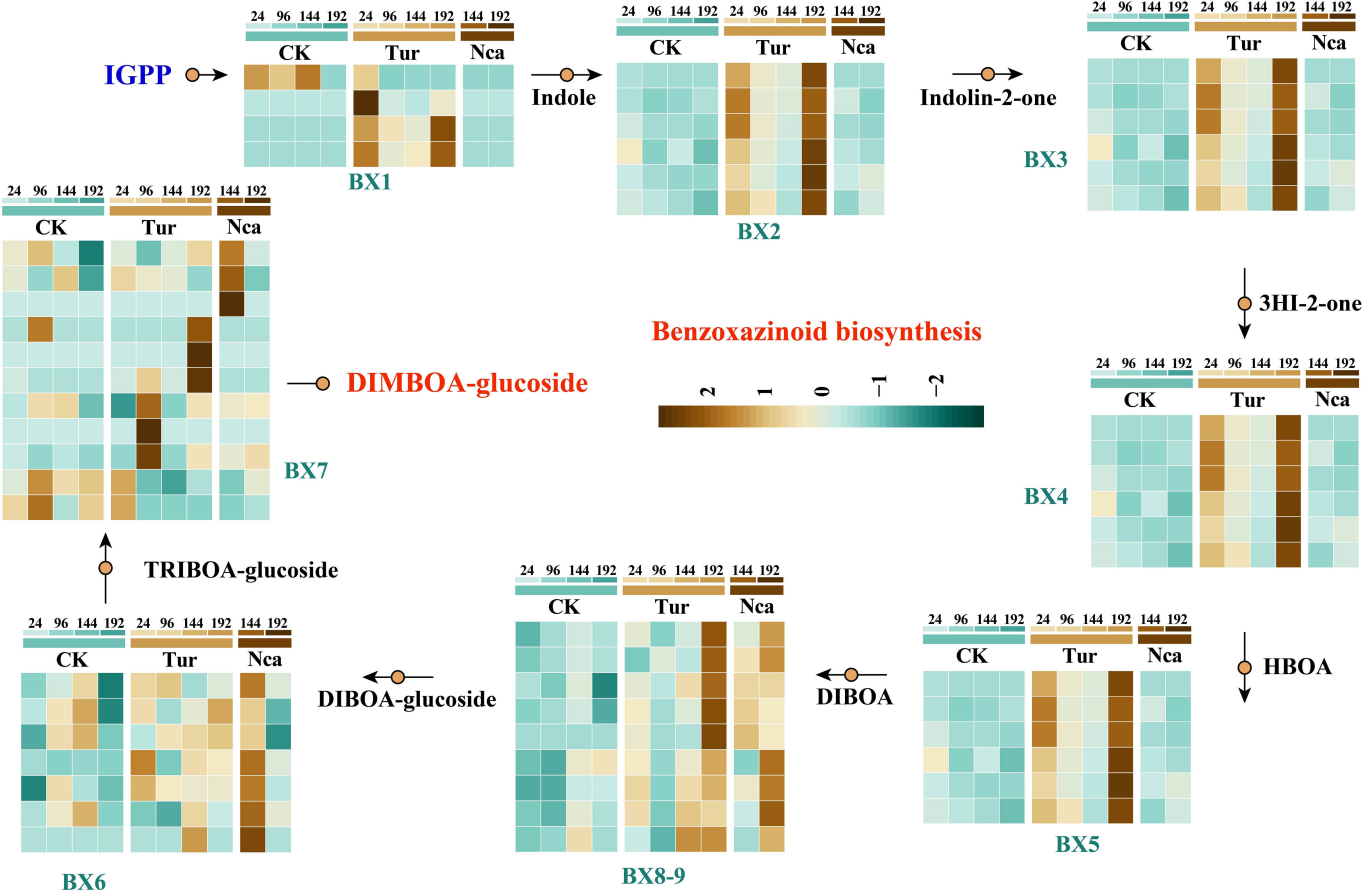
Expression changes of structural genes involved in benzoxazinoid biosynthesis in rose leaves in response to SMI and PMA. Abbreviations are as follows: *BX1* (indole-3-glycerol-phosphate lyase), *BX2* (indole-2-monooxygenase), *BX3* (indolin-2-one monooxygenase), *BX4* (3-hydroxyindolin-2-one monooxygenase), *BX5* (2-hydroxy-1,4-benzoxazin-3-one monooxygenase), *BX6* (2,4-dihydroxy-1,4-benzoxazin-3-one-glucoside dioxygenase), *BX7* (2,4,7-trihydroxy-1,4-benzoxazin-3 -one-glucoside 7-O-methyltransferase), *BX8-9* (UDP-glucosyltransferase).

Benzoxazines are an important family of indole-derived plant metabolites with a variety of insecticidal, antifeedant, antibacterial, and allelopathic properties (Wouters et al., 2016; de Bruijn et al., 2018). The presence of benzoxazines in plant responses to herbivorous insects, particularly in maize, is extensively established. Here, eight structural genes involved in the synthesis of benzoxazines were investigated: *BX1* (indole-3-glycerol-phosphate lyase), *BX2* (indole-2-monooxygenase), *BX3* (indolin-2 -one monooxygenase), *BX4* (3-hydroxyindolin-2-one monooxygenase), *BX5* (2-hydroxy-1,4-benzoxazin-3-one monooxygenase), *BX6* (2,4-dihydroxy-1,4-benzoxazin-3-one-glucoside dioxygenase), *BX7* (2,4,7-trihydroxy-1,4-benzoxazin-3 -one-glucoside 7-O-methyltransferase), and *BX8-9* (UDP-glucosyltransferase) (**Figure 3**). SMI and PMA activated the majority of BX genes, with *BX1*, *BX2*, *BX3*, *BX4*, and *BX5* up-regulated by SMI in Tur24 and Tur96. *BX8-9* was considerably up-regulated in Tur192 and Nca192h, but *BX6* was significantly up-regulated in Nca144. However, SMI or PMA caused up-regulation of *BX7* in Tur24, Tur96, Tur192, and Nca144 (**Figure 3**). These findings suggested that the *BX* gene may play a significant role in response to SMI and PMA in rose, and may also induce potential benzoxazine functions in this response.

When plants are infected by pathogens or exposed to external stimuli, stilbenes are often significantly enhanced (Coutos-Thévenot et al., 2001; Viret et al., 2018). This investigation also discovered four structural genes involved in the production of pterostilbene and pinosylvin: *STS* (pinosylvin synthase), *CYP73A* (trans-cinnamate 4-monooxygenase), *ST* (stilbene synthase), and *ROMT1* (trans-resveratrol di-O-methyltransferase) (**Supplementary Figure 3**). SMI and PMA treatment substantially up-regulated *CYP73A* in Tur24, Tur96, Tur192, Nca144, and Nca192 groups. In the Tur96 group, three *STS*, three *ST*, and two *ROMT1* genes were significantly up-regulated. These findings imply that stilbenes may take a part in rose response to SMI and PMA.

### 3.3 Temporal effects of SMI and PMA on rose leaf metabolome

**Figure 4:**
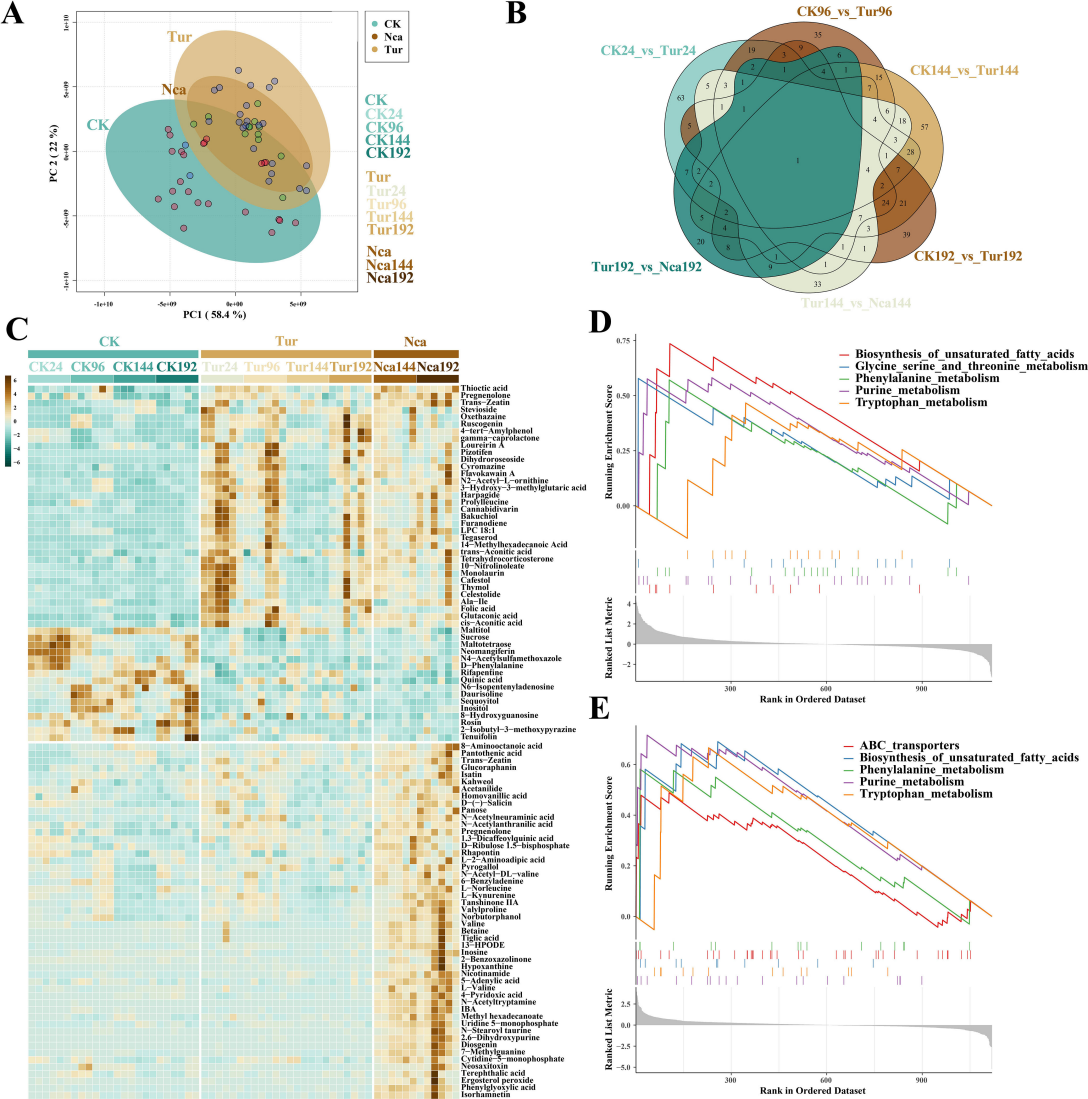
Metabolome overview of the effects of SMI and PMA on rose leaves. **(A)** Metabolome PCA of the effects of SMI and PMA on rose leaves, where different colors represent different treatments. **(B)** Venn diagram of differential metabolites (DIMs) between different groups. **(C)** SMI and PMA treatments induced specific changes in metabolites in rose leaves. The graph presents the 50 most significantly changed DIMs in SMI and PMA groups. **(D)** GSEA of metabolome changes induced by SMI treatment. Lines with different colors represent different metabolites, and the graph presents the most significant five GSEA pathways in SMI groups. **(E)** GSEA of metabolome changes induced by PMA treatment. Lines with different colors represent different metabolites, and the graph presents the most significant five GSEA pathways in PMA groups.

To detect the metabolomic responses of rose to SMI and PMA, control samples from 24 h (CK24), 96 h (CK96), 144 h (CK144), and 192 h (CK192) and SMI-infested groups from corresponding time points (Tur24, Tur96, Tur144, and Tur192) were collected, as well as the metabolome data of rose leaves at 144 h (Nca144) and 192 h (Nca192) of PMA. A total of 2777 metabolites were detected in all samples, of which 1119 metabolites have a distinct chemical class. PCA was used to analyze metabolite level in each replication (**Figure 4A**). The SMI groups were separated from the control groups by a large distance, while the PMA groups fell within the SMI groups but overlapped with the control groups. PC1 separated the majority of control and SMI samples, as well as control and PMA samples, indicating that SMI and PMA induction altered the level of metabolites. In total 174, 158, 227, 149 DIMs were found in Tur24, Tur96, Tur144, and Tur192, respectively, compared with their respective control groups, while 108 and 77 DIMs were discovered in the comparison of Nca144, Nca192 with their SMI counterparts. Venn diagram analysis found 63, 35, 57, and 39 distinct DIMs in Tur24, Tur96, Tur144, and Tur192 respectively, indicating the specificity of rose metabolome alterations at various time periods under SMI treatment (**Figure 4B**). Subsequently, we evaluated the 50 DIMs that altered the most dramatically by SMI and PMA (**Figure 4C**). Biosynthesis of unsaturated fatty acids, glycine serine and threonine metabolism, phenylalanine metabolism, purine metabolism, and tryptophan metabolism were most affected by SMI, according to GSEA (**Figure 4D**). Sucrose, D-Phenylalanine, sequoyitol, LPC 18:1, and trans-Zeatin were among the most important metabolites. In addition, GSEA revealed that DIMs by PMA treatment were mostly implicated in pathways including ABC transporters, biosynthesis of unsaturated fatty acids, phenylalanine metabolism, purine metabolism, and tryptophan metabolism (**Figure 4E**). This showed that PMA induced comparable alterations to SMI in metabolite pathways, but change patterns of each metabolite were different.

Furthermore, we examined the fluctuating patterns of several metabolites in the metabolome (**Supplementary Figure 4**), including steroids, terpenoids, phenol, flavonoids, carbohydrates, and amino acids, as well as an additional six groups. The majority of amino acids and carbohydrates declined dramatically in Tur24, Tur96, and Tur144, but rose in Nca144 and Nca192. These included L-Valine, Ala-Ile, Asp-Glu, L-Serine, D-glucose 1-phosphate, sucrose, and D-ribulose 1,5-bisphosphate. Many steroids and terpenoids, however, started to rise dramatically in the Tur96 group and remained elevated in the Tur144, Tur192, Nca144, and Nca192 groups (**Supplementary Figure 4**). Included among these metabolites were betulin, cyasterone, furanodiene, 4-deacetylneosolaniol, and diacetoxyscirpenol. The up-regulation of these metabolites may provide enhanced capability against SMI. Numerous flavonoids were down-regulated in response to SMI, while the majority of phenol metabolites, including pyrogallol, eugenol, catechol, and 10-Gingerol, were up-regulated in response to PMA.

### 3.4 Trend analysis of transcriptome and metabolome revealed specific response genes and metabolite modules at various time points

**Figure 5:**
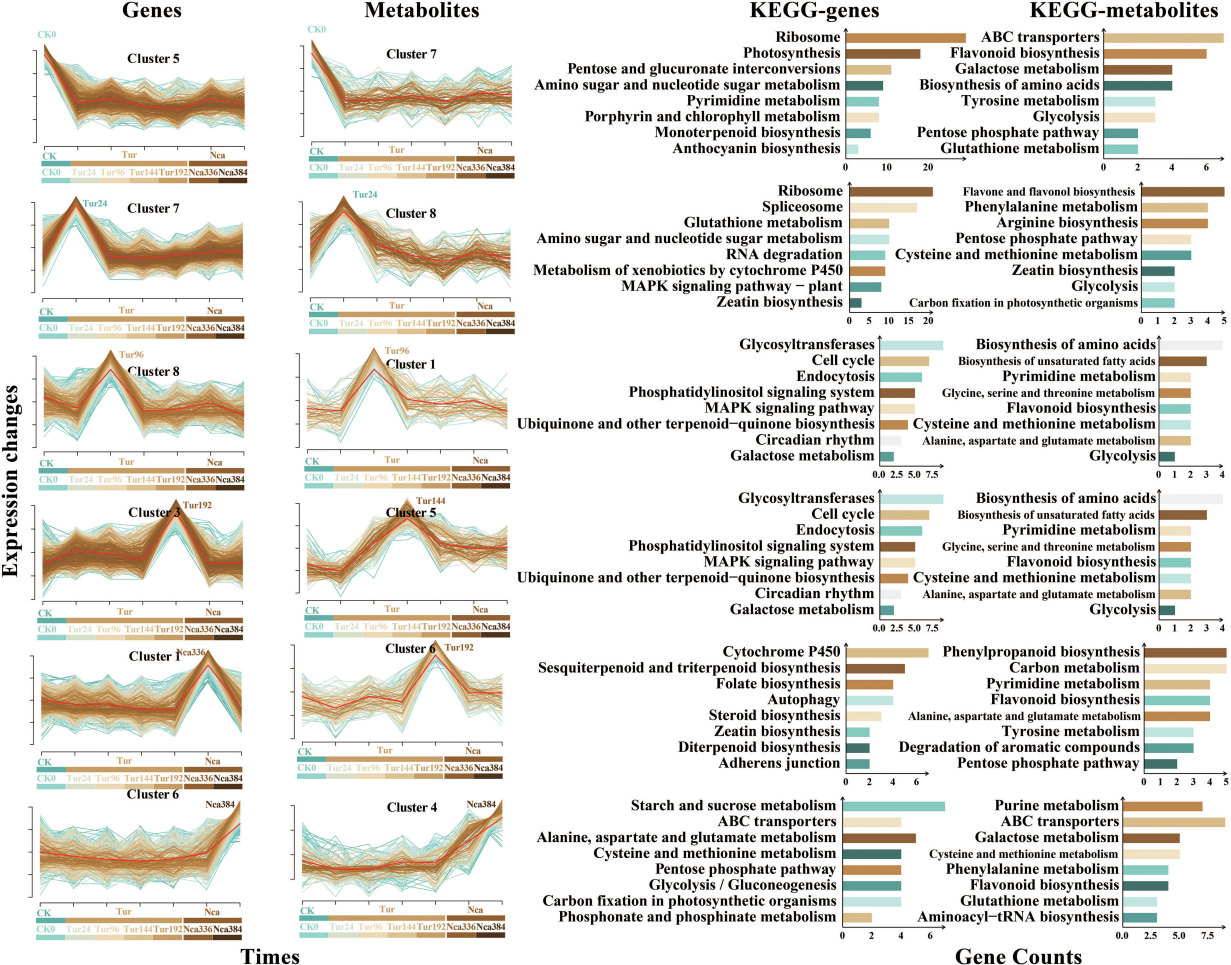
Time series analysis and functional analysis of DEGs and metabolites. Among the four columns in the figure, the first column is the trend analysis of DEGs, and the second is the trend analysis of metabolites. Change trends of the six modules between DEGs and metabolites were basically the same. The abscissa in the figure represents the sampling time, and the ordinate represents the change of genes or metabolites. The third and fourth column represent the KEGG functional enrichment analysis of genes and metabolites in each cluster, respectively. To show the continuity in time, Nca144 is also named Nca336 in figure 8, as 192 h of SMI treatment was followed by 144 h of PMA; Nca192 is also named Nca384, as 192 h of SMI treatment was followed by 192 h of PMA.

To comprehend the dynamics of rose response to SMI and PMA, a brief time-series analysis of total DEGs and metabolites was conducted. All DEGs and metabolites were grouped into eight groups, and six clusters with comparable patterns of transcriptome and metabolome change emerged (**Figure 5**). Cluster5 of the transcriptome and Cluster7 of the metabolome were included in the first change pattern (Module1), with a continual decrease in gene expression and metabolite level by SMI and PMA (**Figure 5**). Functional analysis revealed that Module1 genes were primarily involved in ribosome, photosynthesis, pyrimidine metabolism, and porphyrin and chlorophyll metabolism, whereas Module1 metabolites were primarily involved in ABC transporters, flavonoid biosynthesis, galactose metabolism, and amino acid biosynthesis. This may indicate a rapid reduction in translation in rose leaf cells under SMI, coupled by the weakening of chlorophyll production, pyrimidine metabolism, photosynthesis, and amino acid synthesis, while PMA was unable to appreciably reverse this trend. Cluster7 of the transcriptome and Cluster8 of the metabolome exhibited a particular increase in gene expression and metabolite level with 24 h of SMI, forming the second change pattern (Module2) (**Figure 5**). Functional analysis revealed that Module2 genes were primarily involved in ribosome, spliceosome, metabolism of xenobiotics by cytochrome P450, glutathione metabolism, RNA degradation, MAPK signaling pathway and zeatin biosynthesis, whereas Module2 metabolites were primarily involved in phenylalanine metabolism, zeatin biosynthesis, flavone and flavonol biosynthesis, and arginine biosynthesis. This indicated that 24 h of SMI treatment may rapidly increase the metabolic level of glutathione, boost the synthesis of zeatin, flavones and flavonols, and enhance the activity of cytochrome P450. We speculated that antioxidant-related pathways might predominate in rose leaves in this group. The third pattern (Module3) was a particular increase in gene expression and metabolite level in the Tur96 group, as shown by Cluster8 of the transcriptome and Cluster1 of the metabolome (**Figure 5**). Functional analysis revealed that the Module3 genes were predominantly involved in glycosyltransferases, endocytosis, the MAPK signaling pathway, and galactose metabolism, while the metabolites were predominantly involved in flavonoid biosynthesis, pyrimidine metabolism, biosynthesis of amino acids, ubiquinone and other terpenoidquinone metone biosynthesis, glycine, and tholimonser. This demonstrated that quinone production may be occurring in leaf cells. The production of some amino acids and flavonoids increased as well after 96 h of SMI exposure. The fourth change pattern (Module4) was the particular increase in gene expression and metabolite content at 192 h under SMI, as represented by Cluster3 of the transcriptome and Cluster6 of the metabolome (**Figure 5**). Functional study revealed that the Module4 genes were mostly engaged in the same pathway as Module3 genes, and that the Module4 metabolites were predominantly involved in phenylpropanoid biosynthesis, carbon metabolism, pyrimidine metabolism, and tyrosine metabolism. This suggested that leaf cells were engaged in metabolisms of carbon, pyrimidine, phenylpropanoids, and flavonoids to cope with SMI at 192 h. The fifth pattern (Module5) was the continuing rise in gene expression and metabolite content in Nca336 (To show the continuity in time, Nca144 is also named Nca336 in this part as well as in figure 8, as 192 h of SMI treatment was followed by 144 h of PMA) and Nca384 (To show the continuity in time, Nca192 is also named Nca384 in this part as well as in figure 8, as 192 h of SMI treatment was followed by 192 h of PMA), as represented by Cluster6 of the transcriptome and Cluster4 of the metabolome (**Figure 5**). Functional analysis revealed that Module5 genes were primarily involved in starch and sucrose metabolism, cysteine and methionine metabolism, glycolysis/gluconeogenesis, and carbon fixation in photosynthetic organisms, whereas Module5 metabolites were primarily involved in ABC transporters, aminoacyl-tRNA biosynthesis, and cysteine and methionine metabolism. This demonstrated that rose leaf anabolism began to steadily grow under the PMA treatment, indicating that the plants had gradually escaped from SMI. At 336 h of PMA, Cluster1 of the transcriptome exhibited up-regulated gene expression, primarily linked to Cytochrome P450, sesquiterpenoid and triterpenoid biosynthesis, steroid biosynthesis, and diterpenoid biosynthesis (**Figure 5**). For Module6, at 144 h of SMI and PMA, Cluster5 in the metabolome exhibited a particular increase in metabolite level and was primarily engaged in flavonoid biosynthesis, pyrimidine metabolism, and amino acid biosynthesis (**Figure 5**). A causal association existed between changes in transcriptional gene expression and metabolite content, although there may be a time lag between transcriptome change and resulting changes in metabolome. Changes in gene expression may exert long-lasting impacts on metabolite production, coordinating rose adaptation to SMI and PMA.

### 3.5 Alterations in essential terpenoids and terpenoid synthesis structural genes

**Figure 6:**
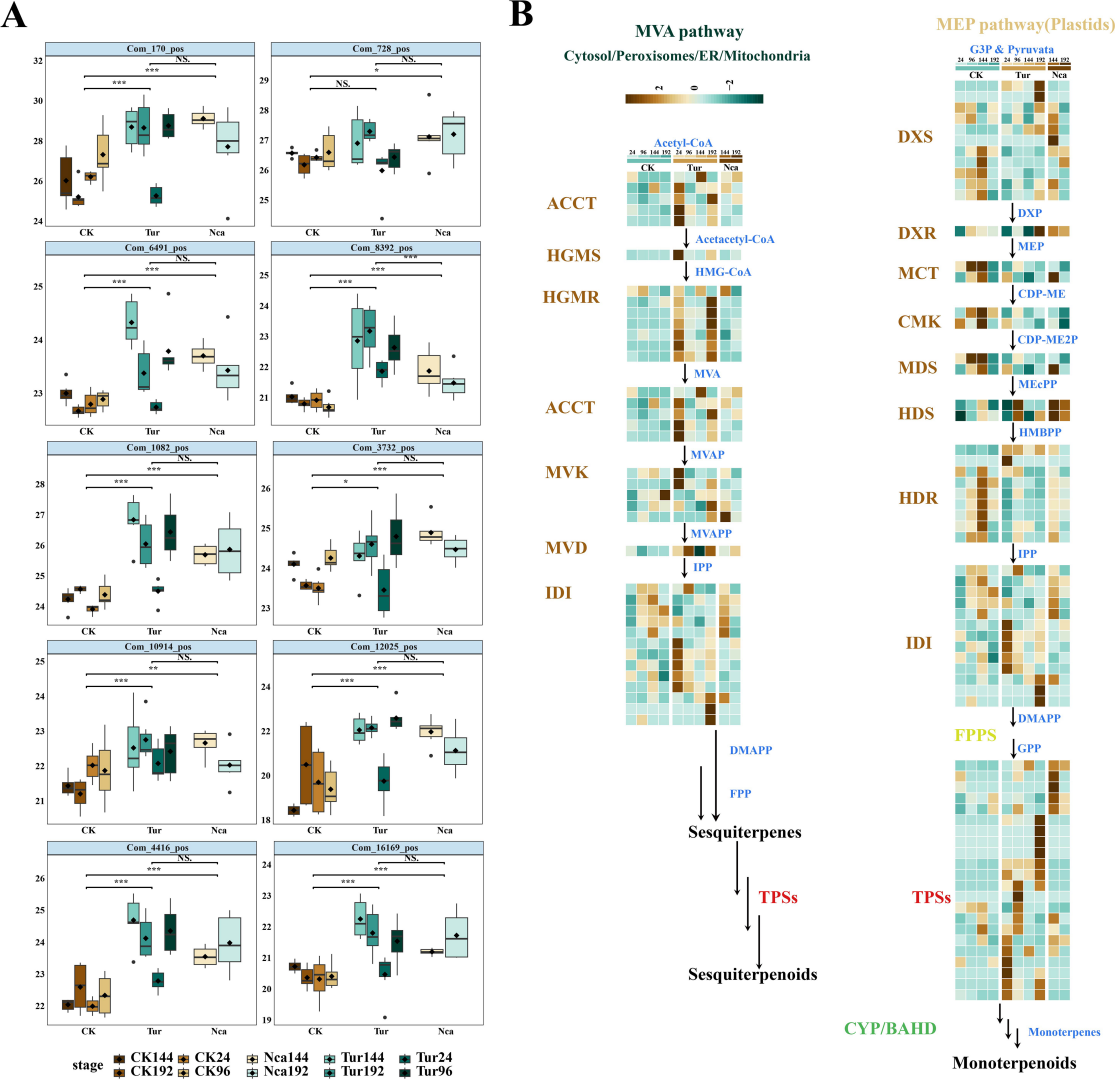
Expression changes of metabolites and structural genes in the terpenoid biosynthetic pathway in rose leaves in response to SMI and PMA. **(A)** Expression changes of 10 terpene metabolites. **(B)** Expression changes of 15 key genes involved in the terpenoid biosynthesis. Structural genes are: acyl-coenzyme A-cholesterol acyltransferase (*ACCT*), hydroxymethylglutaryl coenzyme A synthase (*HGMS*), hydroxymethylglutaryl coenzyme A reductase (*HGMR*), mevalonate kinase (*MVK*), phospho-mevalonate kinase (*PMK*), mevalonate diphosphate decarboxylase (MVD), Deoxy-D-xylulose 5-phosphate synthase (*DXS*), 1-deoxy-D-xylulose 5-phosphate reductoisomerase (*DXR*), 2-C-methyl-D-erythritol-4-phosphate cytidylyltransferase (*MCT*), 4-(cytidine -5-diphospho)-2-C-methyl-D-erythritol kinase (*CMK*), 2-C-methyl-D-erythritol-2,4-cyclodiphosphate synthase (*MDS*), (E)-4-hydroxy-3- methyl-but-2-enyl-pyrophosphate synthase (*HDS*), (E)-4-hydroxy-3-methyl-but-2-enyl-pyrophosphate reductase (*HDR*) and isopentenyl diphosphate isomerase (*IDI*), lavandulyl diphosphate synthase (*LPPS*). * P < 0.05, ** P < 0.01, *** P < 0.001, ns: not significant. Student’s t test.

Phytoalexins, primarily involved in pathways related to sesquiterpenes, flavonoids, isoflavonoids and phenylpropanoid compounds, play a crucial role in biotic stress tolerance. At 96 h of SMI, 10 terpene metabolites, including ganoderiol A, citral, bakuchiol, betulin, furanodiene, Thymol, 4-Deacetylneosolaniol, cyasterone, ruscogenin, cannabidivarin, diacetoxyscirpenol, and forskolin, were considerably up-regulated, and this trend remained in Nca144 and Nca192 (**Figure 6A**). This indicated that terpenoids play an important role in rose response to SMI and PMA, so 15 key genes involved in the terpenoid biosynthesis were subsequently analyzed. These genes included acyl-coenzyme A-cholesterol acyltransferase (*ACAT*), hydroxymethylglutaryl coenzyme A synthase (*HMGS*), hydroxymethylglutaryl coenzyme A reductase (*HMGR*), meval (cytidine -5-diphospho) -2-C-methyl-D- erythritol kinase (*CMK*), 2-C-methyl-D-erythritol-2,4-cyclodiphosphate synthase (*MDS*), (E)-4-hydroxy-3-methyl-but-2-enyl-pyrophosphate synthase (*HDS*), and (E)-4-hydroxy-3-methyl-but-2-enyl-pyrophosphate reductase (*LPPS*). The research revealed that the essential genes *ACCT*, *HGMS*, *HGMR*, *MVK*, *MVD*, *IDI*, and *TPSs* in the MVA pathway were all up-regulated in response to SMI and PMA, indicating that the MVA pathway was active in rose response to SMI and PMA. Six essential genes in the mechanical, electrical and plumbing (MEP) system, including *DXS*, *DXR*, *MCT*, *CMK*, *MDS*, and *HDR*, did not exhibit an increasing trend of expression throughout the Tur and Nca treatments (**Figure 6B**). These data revealed that the MVA route was the primary synthesis pathway in response to SMI and PMA, and that sesquiterpenoids may be the primary terpenoids in this response.

### 3.6 Characterization of vital transcription factors in response to SMI and PMA in rose

Transcription factors play a crucial role in biotic stress resistance. The key transcription factors in related external stimuli included DRE1C, BH035, MYB14, ERF110 and WRKY24, and those involved in JA biosynthesis, such as LOX31, LOX21, and LOX15 (**Supplementary Figures 5A, B**). This demonstrated that PMA caused rose leaves to recover from the SMI condition and resume regular development. The 80 genes whose expression was restored at 192 h of PMA (Nca192) were mostly associated with carboxylic acid metabolism, oxidoreductase activity, and lipid metabolism. These transcription factors included BH006, WRK27, MYC2, NAC71, MY108, COMT1, AAT2, PAT1, and those involved in the synthesis of secondary metabolites (**Supplementary Figures 5C, D**).

**Figure 7:**
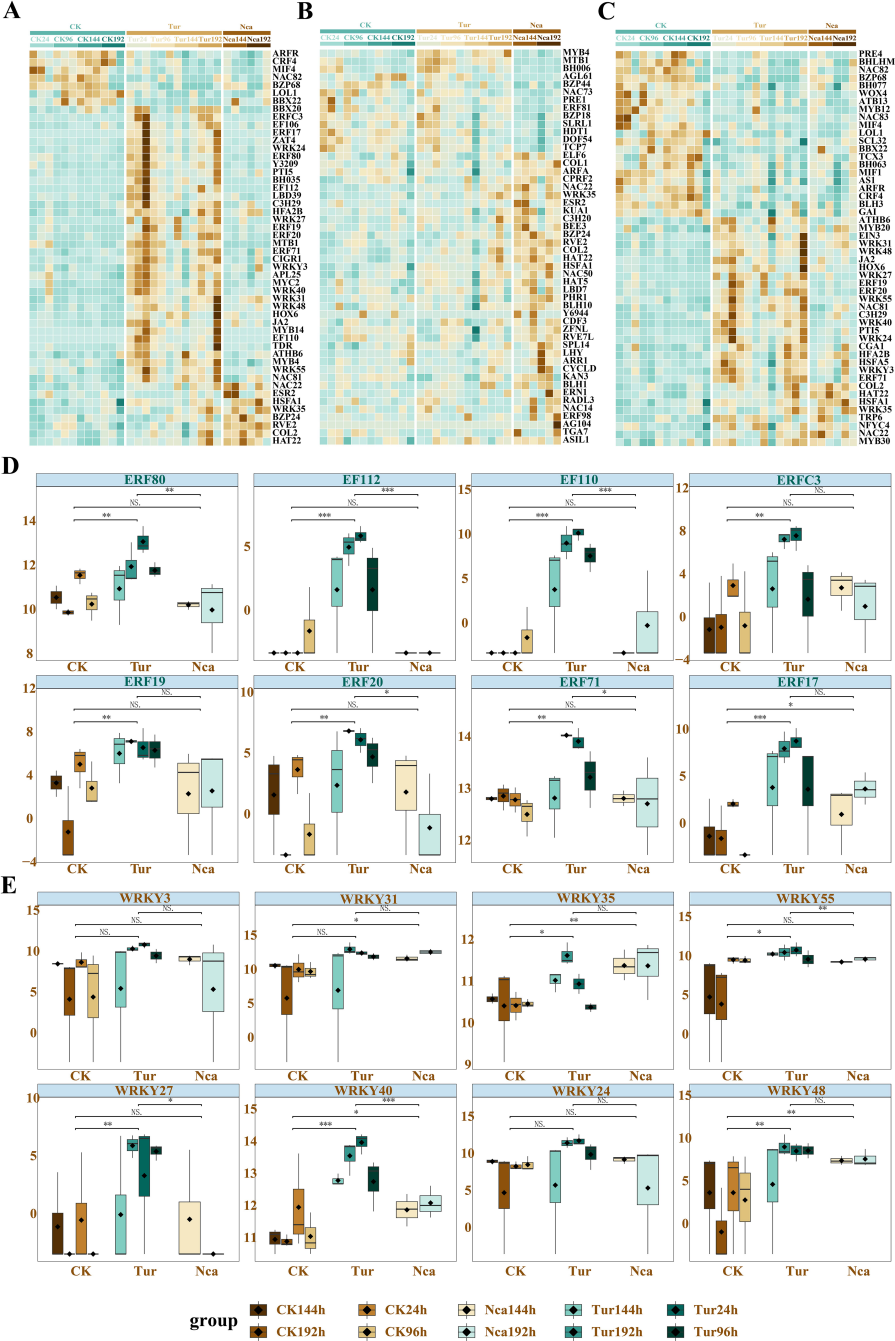
Identification of key transcription factors responding to SMI and PMA in rose. **(A)** The 50 transcription factors with the most dramatic alteration among SMI, PMA, and control groups. **(B)** The 50 transcription factors with the most dramatic alteration between *N. californicus*-treated (PMA) and non *N. californicus*-treated groups (SMI and control). **(C)** Eight members of the ERF transcription factor family were up-regulated by SMI and down-regulated by PMA. **(D)** The 50 transcription factors with the most dramatic alteration between *T. urticae*-infested (SMI and PMA) and non *T. urticae*-infested (control) groups. **(E)** Eight members of the WRKY transcription factor family were up-regulated by SMI and down-regulated by PMA. * P < 0.05, ** P < 0.01, *** P < 0.001, ns: not significant. Student’s t test.

Studies have shown the essential roles of WRKY and NAC transcription factor families in the regulation of plant defense against insect herbivores (He et al., 2020; Kundu and Vadassery, 2021). Therefore, we identified the 50 transcription factors in SMI and PMA groups that altered the most dramatically (**Figures 7A-C**), including a considerable number of WRKY and ERF transcription factor family members. CRF4, MIF4, NAC82, BZP68, LOL1, BBX22 were identified as negative regulators responding to SMI. Eight members of the ERF family, ERF80, EF112, EF110, ERFC3, ERF19, ERF20, ERF71, and ERF17, were considerably up-regulated in response to SMI and down-regulated by PMA (**Figure 7D**). This may suggest that these eight transcription factors were important regulators in response to SMI and PMA. In addition, the WRKY family genes *WRKY3*, *WRKY31*, *WRKY35*, *WRKY55*, *WRKY27*, *WRKY40*, *WRKY24*, and *WRKY48* exhibited the same change pattern as the ERF family genes (**Figure 7E**). Among these, the variation of *WRKY40* was the most notable, which suggested that it may be a potential regulator of rose response to biotic stress. The validation of these trascription factors and structural genes were conducted by QRT-PCR (**Supplementary Figure 6**).

## 4 Discussion

Spider mites are one of the primary pests of greenhouse roses, inflicting extensive leaf damage throughout the growth season. The current understanding of rose defenses in response to *T. urticae* attack is limited. This study provides comprehensive insights into the molecular mechanisms underlying the responses of roses to red spider mite infestation (SMI) and the antagonistic actions of predatory mites (PMA). Our findings revealed significant changes in the transcriptome and metabolome of rose leaves within 24 hours of SMI, indicating a rapid activation of defense-related pathways. Previous research has shown that COPB2 and the Dof protein may play a significant role in reaction to SMI in rose (Nan et al., 2021; Lee et al., 2022). However, no comprehensive investigation has explored the molecular mechanism underlying these defensive reactions. Plant transcriptional responses to insect assaults are a time-dependent, dynamic, and complicated process involving signaling and synthetic reprogramming. Previous studies of plant transcriptome responses to herbivores have focused on just one single induction time point (Yang et al., 2015; Wang et al., 2017), which may not be enough to reveal the full-depth profile of temporal patterns of gene activity during plant-insect interactions. It is essential to analyze this interaction at different time points during infestation. In this work, the DEGs in the dynamic alterations of the transcriptome decreased with SMI time extended, and the defensive response peaked at 24 h of SMI. The metabolic response was not consistent with that of the transcriptome, as most defensive metabolites, including terpenes, stilbenes, and sterols, were highly up-regulated at 96 h under SMI. This may imply that SMI first induced the expression of metabolic pathway-related genes at 24 h of SMI, which contributed to the accumulation of defensive chemicals accordingly that were finally detected at 96 h.

Numerous phytophagous species, like *T. urticae*, have developed characteristics that allow them to circumvent plant defenses via three primary strategies: avoidance, metabolic resistance, and suppression (Kant et al., 2015; Blaazer et al., 2018; Snoeck et al., 2018). After infestation with *T. urticae*, JA biosynthesis-related genes, such as *LOX31*, *LOX21*, and *LOX15*, were significantly up-regulated. Nonetheless, metabolic data revealed that key hormones involved in plant biological defense, such as JA, SA, and ABA, went through no substantial changes. In addition, no significant changes were observed in the expression of key genes associated with the JA, SA, or ABA response in rose, indicating that the phytohormone-mediated defense in rose was considerably repressed. Phytophagous mites have developed specific chemicals to inhibit plant defense responses (Carrillo et al., 2011; Grbic et al., 2011; Snoeck et al., 2018; Wybouw et al., 2018).

Insects are repelled by defensive chemicals produced by plants. Examples of these chemicals in this category include benzoxazines, sesquiterpenes, flavonoids, isoflavonoids, phenylpropanes, and stilbenes (Liu et al., 2006; Treutter, 2006; Habash et al., 2020; Valletta et al., 2021; Luo et al., 2022). They have insecticidal, antifeedant, antibacterial, and allelopathic properties. Terpenoids, such as ganoderiol A, citral, bakuchiol, betulin, furanodiene, thymol, 4-deacetylneosolaniol, cyasterone, ruscogenin, cannabidivarin, diacetoxyscirpenol, and forskolin, started to accumulate in substantial amount after infested by *T. urticae*. These terpenoids are pivotal volatiles that not only reduce the feeding rate of spider mites, but also attract their predators (Block et al., 2019; Boncan et al., 2020). The synthesis of these defense-related metabolites and the regulation of their gene expression play an important role in plant’s resistance to external invasion. Key pathways such as sesquiterpenoid and triterpenoid biosynthesis, benzoxazinoid biosynthesis, and the MAPK signaling pathway were notably enriched. These pathways are known to play crucial roles in plant defense against biotic stress. The increased expression of genes and metabolites associated with phytosterol biosynthesis, the mevalonate (MVA) pathway, benzoxazinoid biosynthesis, and stilbenoid biosynthesis highlights the complex biochemical network roses utilize to mount an effective defense response against SMI. Simultaneously, the expression of phytosterol biosynthesis-related genes was up-regulated upon *T. urticae* infestation, and the amount of stigmasterol increased rapidly after 96 h of SMI. As important components of the cell membrane and lipid raft, phytosterols are directly associated with membrane stability (Fakih et al., 2018; Du et al., 2022). To maintain membrane integrity and lipid raft function, plants primarily react to adversity by modifying the relative sterol concentration (Ras et al., 2013). Phytosterol-related genes, such as *SQS*, *SMT2*, and *C22*-sterol desaturase, are implicated in the immune response to bacterial infections. By raising phytosterol levels, rose may cope with the SMI-caused instability of cell membrane structure, and withstand the harm brought by exogenous chemicals. Interestingly, the rose response to PMA diminished over time, with significant recovery of organic acid and lipid metabolic levels after 192 hours. This suggests that while PMA is effective in the short term, the long-term resilience of roses may require additional interventions or sustained biocontrol strategies.

In response to arthropod feeding, plants react with a depolarization of the membrane potential (Vm), accompanied by a rise in cytosolic Ca^2+^ and ion channel activity, as well as a burst of reactive oxygen species (ROS) and reactive nitrogen species (RNS). In addition, a number of studies have shown that *T. urticae* infestation causes cell damage that results in changes in cytosolic Ca^2+^ levels and Vm fluctuations. In conjunction with ROS production is an accumulation of phenolics in the wounding areas of plant species after pathogen attack (Leitner et al., 2005; Santamaria et al., 2012; Santamaría et al., 2017). It has been found that barrelclover (*Medicago truncatula*) and thale cress plants accumulate ROS and phenolic chemicals at the locations of *T. urticae*-caused injury. ROS molecules, notably H_2_O_2_, are vital for defensive signaling, regulation of cell proliferation, and enhancement of biological functions through oxidative pathways (Mittler, 2017). In rose leaves, the expressions of genes such as *GSTX3*, *GSTX6*, *GSTX1*, and *GSTXC* were up-regulated at 96 h of SMI, but not at 24 h. In their enzymatic ROS scavenging mechanism, *GST* genes play a crucial role (Anderson and Davis, 2004; Lan et al., 2009). This revealed that the accumulation of ROS between 24-96 h of SMI induced the particular up-regulation of the *GST* genes to avoid cell damage from ROS. Similarly, the expression of catalase in rose leaves under SMI was similarly up-regulated, which was harnessed as a strategy in rose to combat the ROS outbreak (Mhamdi et al., 2010; Sofo et al., 2015).

The role of PMA in mitigating the effects of SMI was evident through the progressive recovery of roses, as indicated by the restoration of gene expression and metabolite levels. PMA treatment led to the reversion of roses to their normal growth state by reversing the damage caused by SMI. Genes from the WRKY, ERF, and NAC transcription factor families play a significant role in plant growth and stress responses (Nakashima et al., 2007; Jiang et al., 2017; Erpen et al., 2018). Numerous positive and negative regulators involved in rose responses to SMI and PMA were found in this research. ARFR, CRF4, MIF4, NAC82, BZP68, LOL1, and BBX22 were identified as negative regulators of the rose response to SMI, which started to be gradually down-regulated at 24 h of SMI. ARFR was engaged in the early rose response to auxin and may mitigate the inhibition effects of SMI on plant growth or metabolism. Meanwhile, multiple WRKY and ERF family transcription factors have been identified as positive regulators in response to SMI. These transcription factors include ERF80, EF112, EF110, ERFC3, ERF19, ERF20, ERF71, ERF17, WRKY3, WRKY31, WRKY35, WRKY55, WRKY27, WRKY40, WRKY24, and WRKY48. Notably, the restoration of 190 essential genes, including critical transcription factors such as DRE1C, BH035, MYB14, EF110, WRKY24, NAC71, and MY108, underscores the pivotal role of these genes in the recovery process. The expression of these transcription factors was consistently increased in Tur24 but decreased in Nca144. This suggested that these transcription factors might function as regulators in the interactions among rose, *T. urticae*, and *N. californicus.*

Overall, this study sheds light on the intricate interactions between roses, T. urticae, and N. californicus. By elucidating the transcriptome and metabolome changes associated with SMI and PMA, our research provides valuable insights into the molecular basis of plant defense and recovery. These findings have practical implications for developing sustainable pest management strategies in greenhouse cultivation, enhancing the resilience of roses against mite infestations while minimizing chemical pesticide use. Future studies should focus on the long-term effects of PMA and explore additional biocontrol agents or combined approaches to ensure prolonged protection and optimal growth of roses. Understanding the temporal dynamics of rose responses to biotic stress and biocontrol interventions will be crucial for developing integrated pest management practices that are both effective and environmentally friendly.

## 5 Conclusions

Our study revealed significant insights into the defensive responses of greenhouse-grown roses to SMI and the protective mechanisms provided by PMA. Transcriptome and metabolome analyses showed that 24 h of SMI caused substantial changes in defense-related genes and metabolites. Key pathways involved included terpenoid biosynthesis, MAPK signaling, and phenylpropanoid biosynthesis. PMA facilitated recovery from SMI, restoring the expression of essential genes and allowing roses to revert to their normal growth state. These findings underscore the potential of PMA as an effective strategy for reducing SMI damage in roses. Our results enhance the understanding of plant defense mechanisms and predator-mediated protection, providing valuable insights for future pest management strategies in the rose industry.

## Data Availability

The datasets presented in this study can be found in online repositories. The names of the repository/repositories and accession number can be found below: https://www.ncbi.nlm.nih.gov/, PRJNA1134402.
