# Supplementary figures and images for: Temporal transcriptome and metabolome study revealed molecular mechanisms underlying rose responses to red spider mite infestation and predatory mite antagonism
Source: Front Plant Sci. 2024 Aug 14;15:1436429. doi: 10.3389/fpls.2024.1436429 (PMC11368075; doi:10.3389/fpls.2024.1436429)

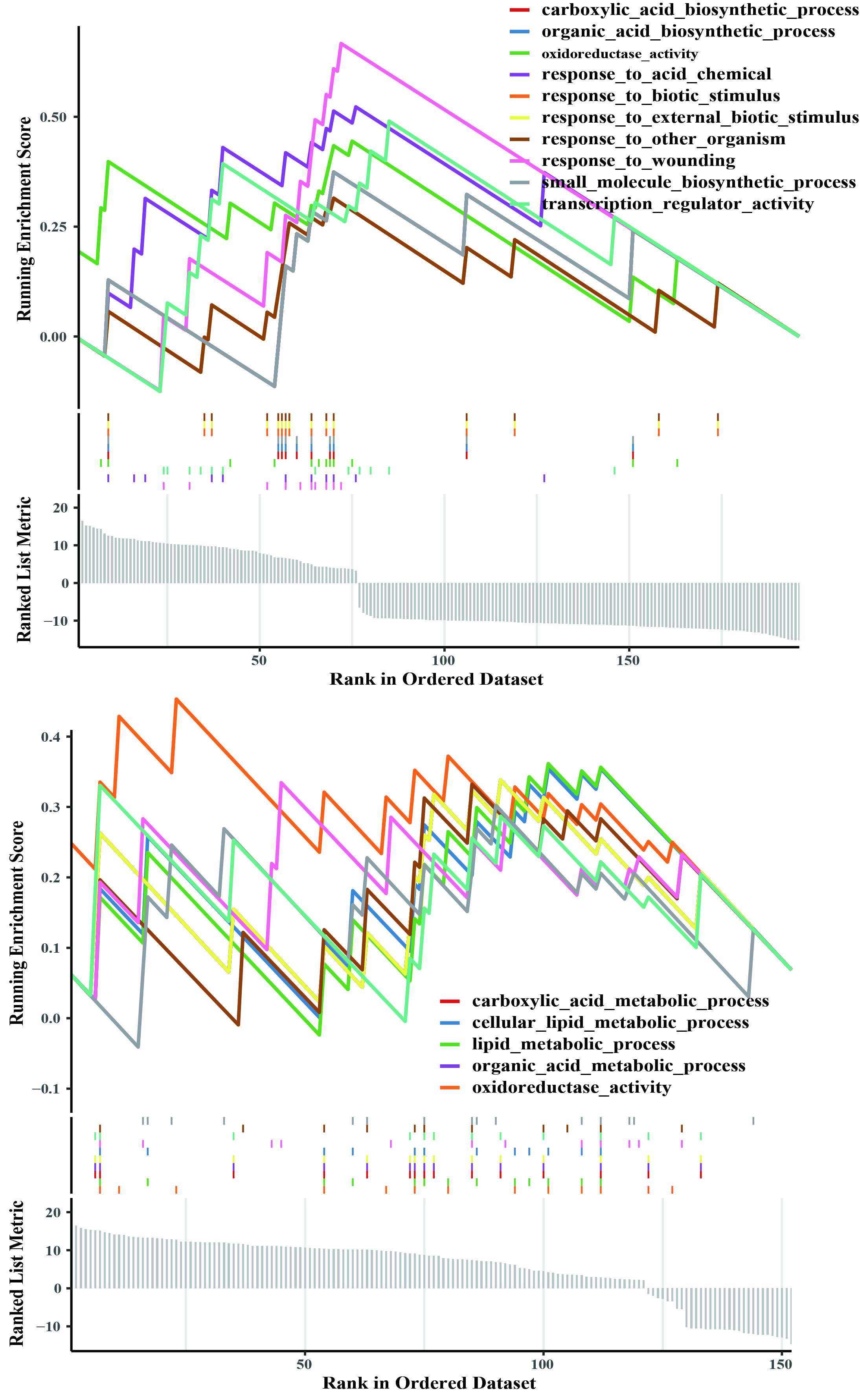

Supplement: Supplementary file 1 [file Image1.jpeg]

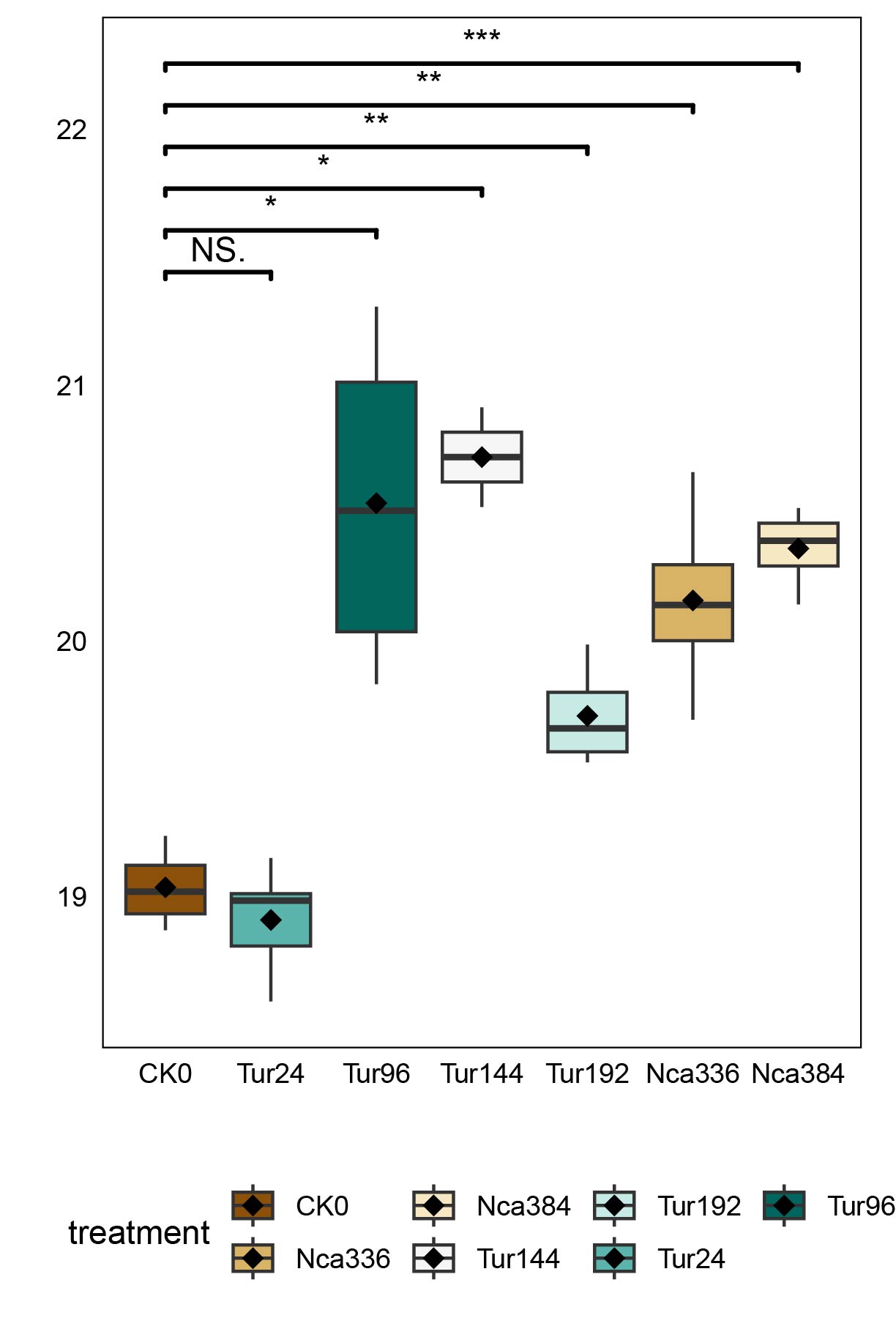

Supplement: Supplementary file 2 [file Image2.jpeg]

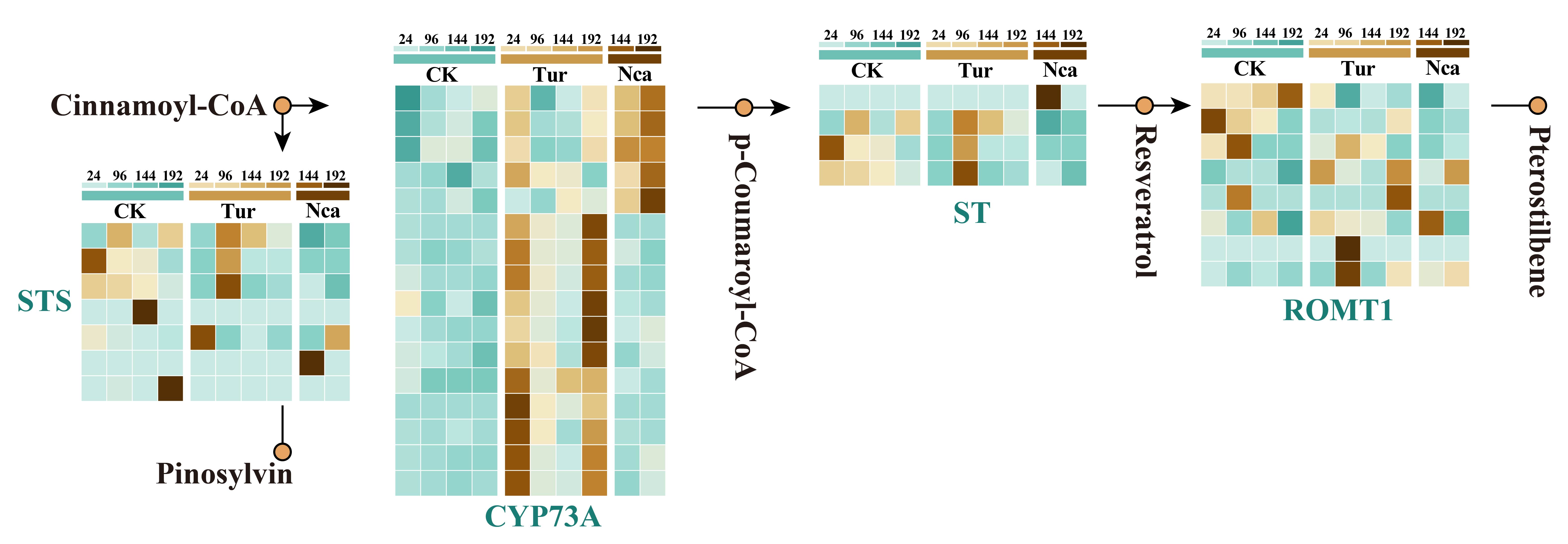

Supplement: Supplementary file 3 [file Image3.jpeg]

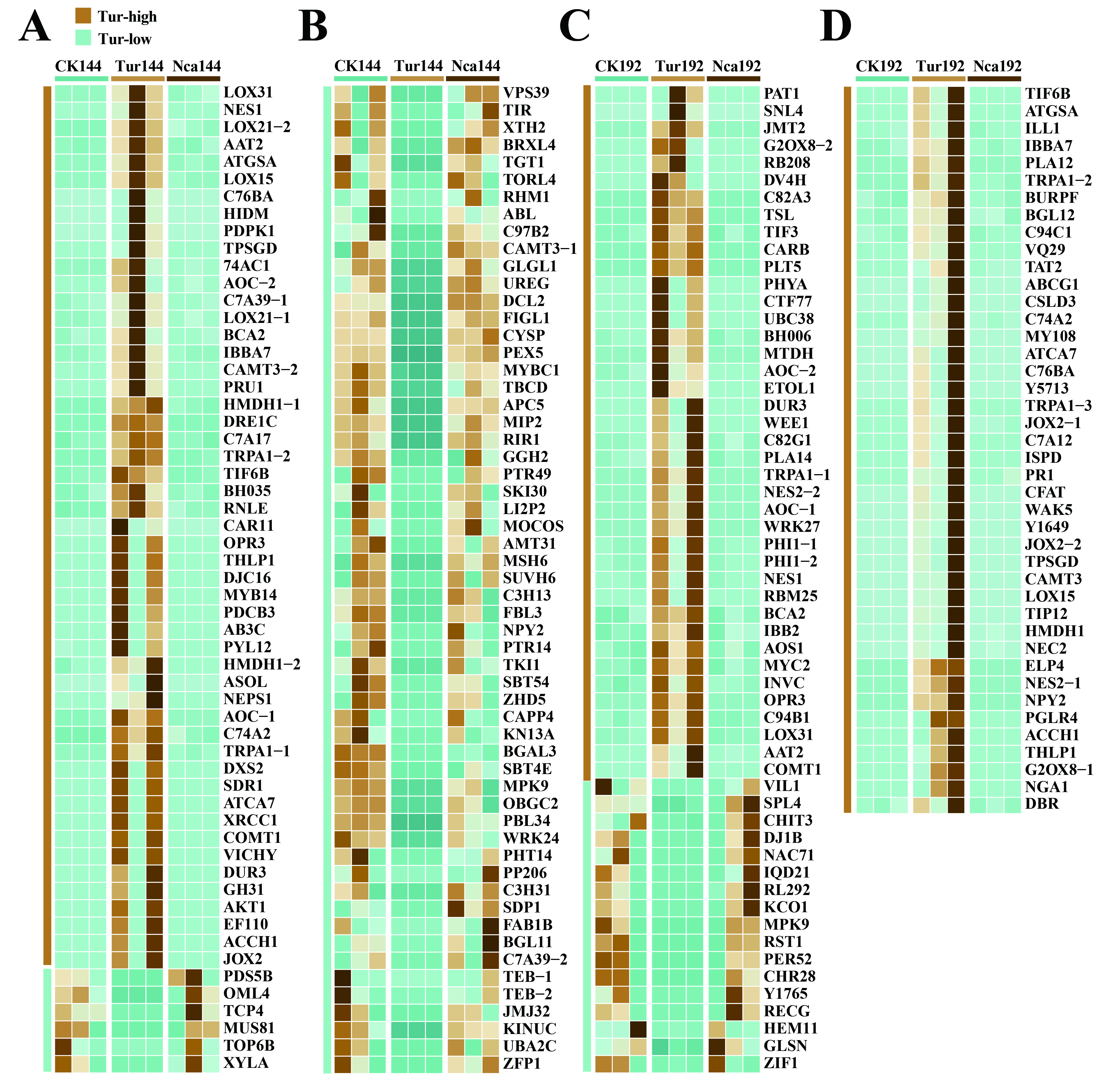

Supplement: Supplementary file 5 [file Image5.jpeg]

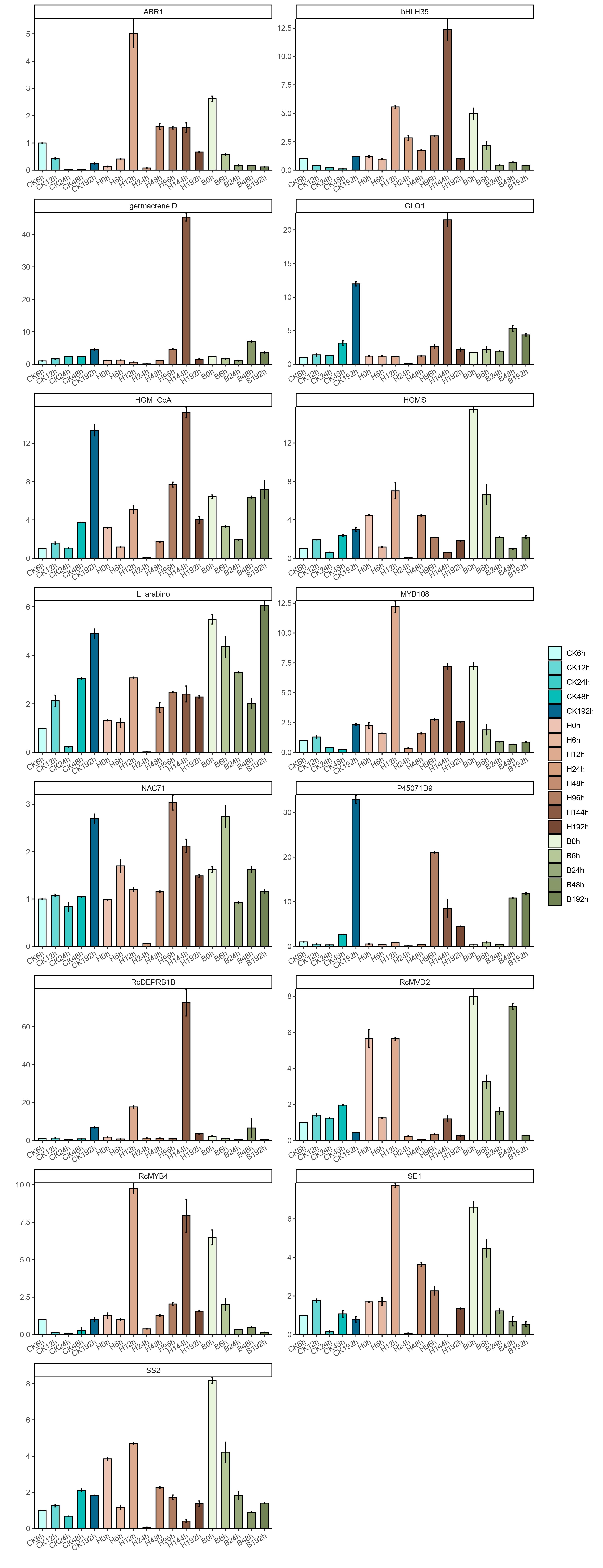

Supplement: Supplementary file 6 [file Image6.jpeg]
