# Supplementary material for: Temporal transcriptome and metabolome study revealed molecular mechanisms underlying rose responses to red spider mite infestation and predatory mite antagonism
Source: Front Plant Sci. 2024 Aug 14;15:1436429. doi: 10.3389/fpls.2024.1436429 (PMC11368075; doi:10.3389/fpls.2024.1436429)
Supplement: Supplementary file 7 [file Table1.docx]

**Supplementary Figure 1** GSEA of genes whose expression was restored by 144 h and 192 h of PMA. Lines with different colors represent different genes, and the graph presents the most significant 10 GSEA pathways in the group.

**Supplementary Figure 2** Alterations of the stigmasterol level at different time points of SMI and PMA in rose

**Supplementary Figure 3** Expression changes of structural genes involved in stilbene biosynthesis in rose leaves in response to SMI and PMA. The four key structural genes involved are *STS* (pinosylvin synthase), *CYP73A* (trans-cinnamate 4-monooxygenase), *ST* (stilbene synthase), and *ROMT1* (trans-resveratrol di-O-methyltransferase).

**Supplementary Figure 4** Changes of various metabolites in rose leaves in response to SMI and PMA. These include six categories, including steroids, terpenoids, amino acids, phenol, carbohydrates and flavonoids.

**Supplementary Figure 5** PMA restored expression of abnormally expressed genes caused by SMI in rose leaves. A, B: Hot spots of genes whose expression was restored by 144 h of PMA treatment. Brown represents up-regulated DEGs by SMI, and cyan represents down-regulated DEGs by SMI. C, D: Hot spots of genes whose expression was restored 192 h under PMA. Brown represents up-regulated DEGs by SMI, and cyan represents down-regulated DEGs by SMI.

**Supplementary Figure 6** Expression of transcription factors and structural genes verified by qRT-PCR.
