# Supplementary material for: Temporal transcriptome and metabolome study revealed molecular mechanisms underlying rose responses to red spider mite infestation and predatory mite antagonism
Source: Front Plant Sci. 2024 Aug 14;15:1436429. doi: 10.3389/fpls.2024.1436429 (PMC11368075; doi:10.3389/fpls.2024.1436429)
Supplement: Supplementary file 8 [file Table2.docx]

Supplementary Table 1 Primers for quantitative real time polymerase chain reaction (qRT-PCR)

| **gene** | **forward sequence(5' to 3')** | **reverse sequence(5' to 3')** |
| --- | --- | --- |
| RcMVD2 | AATCAAGGGCCAGCAGTTCA | GCCCAGTTTCAGGGTTGAGT |
| RcMYB4 | GAAGAGTTGCAGGCTGAGGT | CGCAATCGCAGACCATTTGT |
| RcDREBP 1B | TCTGCGATGCTTCTGCTTCT | ATGTGGGTTAGGGTTTGCCC |
| Rc-bHLH35 | CAGTGGTGGTGAGCTTGACA | CCTCCCAGACAAAGCAGTGAT |
| Rc-MYB108 | GGACTCGTTTGGGACTCAGG | TGGTGATGAGGATTGGTGGC |
| Rc-GLO1 | AGCATTAGCATTGGGTGCCT | AGCTCAAACTCCTCACGCAA |
| RcABR1 | CCCGTGGACTATGAGGAAGC | ACAACATAGACGGCGACACA |
| Rc-SE1 | GCAAAGATGGGTGCAAGTCC | TCAATCACATGCACTTGGCG |
| Rc-NAC71 | AGCTCCAAGAGGCACCAATC | TGGAAACTCGTACTGGGGGA |
| Rc-HGMS | TGGGATGGACGCTATGGACT | TCAGGTCCAATCAGCAAGGC |
| Rc-L-arabino | GTGAGTTCCTGATCCGCCTC | ACCTCTTTGCGGGATCTGTG |
| Rc-p450 71D9 | TTGGCTGTCTACCCCATCAC | CCCACTCTGCTGACGAAACA |
| Rc-SS2 | AGTGGCATTTTGCATGTGGT | GCCTCCTGATAACCCTTCCC |
| Rc-HMG-COA | CAAGTCGGTGGTCTGTGAGG | AGCAATGGCGGAACCAGTAA |
| Rc-germacrene D | TAACTTTGCATGTGCCACGG | TATACAGGCACGGGTTGGAC |

Supplementary table 2 Overview of sequencing data

| Sample | Raw Reads | Clean Reads | Raw Base(G) | Clean Base(G) | Effective(%) | Error(%) | Q20(%) | Q30(%) | GC(%) |
| --- | --- | --- | --- | --- | --- | --- | --- | --- | --- |
| B-144h-1 | 41472824 | 40584712 | 6.22 | 6.09 | 97.86 | 0.03 | 96.89 | 91.48 | 45.69 |
| B-144h-2 | 47029374 | 45856470 | 7.05 | 6.88 | 97.51 | 0.03 | 96.8 | 91.32 | 45.27 |
| B-144h-3 | 45804832 | 44938526 | 6.87 | 6.74 | 98.11 | 0.03 | 97.17 | 92.12 | 45.29 |
| B-192h-1 | 43687822 | 42554904 | 6.55 | 6.38 | 97.41 | 0.03 | 96.97 | 91.58 | 43.6 |
| B-192h-2 | 48522292 | 47522958 | 7.28 | 7.13 | 97.94 | 0.03 | 97.08 | 91.98 | 44.72 |
| B-192h-3 | 45432552 | 44676708 | 6.81 | 6.70 | 98.34 | 0.03 | 97.35 | 92.69 | 45.93 |
| CK-144h-1 | 47355968 | 46467086 | 7.10 | 6.97 | 98.12 | 0.03 | 97.1 | 92.03 | 45.86 |
| CK-192h-2 | 45655674 | 44175190 | 6.85 | 6.63 | 96.76 | 0.03 | 97.02 | 91.84 | 45.99 |
| CK-24h-2 | 42543704 | 41275656 | 6.38 | 6.19 | 97.02 | 0.03 | 96.84 | 91.39 | 44.82 |
| CK-96h-2 | 46271980 | 45295190 | 6.94 | 6.79 | 97.89 | 0.03 | 96.77 | 91.4 | 44.95 |
| H-144h-1 | 48520236 | 47808988 | 7.28 | 7.17 | 98.53 | 0.03 | 97.5 | 92.95 | 45.91 |
| H-144h-2 | 43126068 | 41623320 | 6.47 | 6.24 | 96.52 | 0.03 | 97.02 | 91.67 | 42.28 |
| H-144h-3 | 47135252 | 46345950 | 7.07 | 6.95 | 98.33 | 0.03 | 96.96 | 91.67 | 44.16 |
| H-192h-1 | 43149258 | 41269990 | 6.47 | 6.19 | 95.64 | 0.03 | 96.89 | 91.46 | 44.07 |
| H-192h-2 | 45531242 | 43876362 | 6.83 | 6.58 | 96.37 | 0.03 | 97.14 | 92.08 | 45.33 |
| H-192h-3 | 47022506 | 45791860 | 7.05 | 6.87 | 97.38 | 0.03 | 96.99 | 91.76 | 44.52 |
| H-24h-3 | 47671778 | 47027176 | 7.15 | 7.05 | 98.65 | 0.03 | 97.19 | 92.34 | 46.58 |
| H-24h-1 | 41896480 | 40974446 | 6.28 | 6.15 | 97.8 | 0.03 | 96.93 | 91.6 | 45.66 |
| H-24h-2 | 46282600 | 45042510 | 6.94 | 6.76 | 97.32 | 0.03 | 97.14 | 92.09 | 44.8 |
| H-96h-1 | 44049824 | 43354026 | 6.61 | 6.50 | 98.42 | 0.03 | 97.19 | 92.22 | 44.45 |
| H-96h-2 | 40823114 | 40069104 | 6.12 | 6.01 | 98.15 | 0.03 | 96.86 | 91.43 | 44.48 |
| H-96h-3 | 45713984 | 44765228 | 6.86 | 6.71 | 97.92 | 0.03 | 96.94 | 91.63 | 44.85 |
| CK-144h-2-1 | 48065452 | 47321564 | 7.21 | 7.10 | 98.45 | 0.03 | 97.26 | 92.36 | 45.54 |
| CK-144h-2-2 | 46075964 | 45146016 | 6.91 | 6.77 | 97.98 | 0.03 | 96.96 | 91.69 | 46.14 |
| CK-192h-1-1 | 43306294 | 42249020 | 6.50 | 6.34 | 97.56 | 0.03 | 97.08 | 91.9 | 45.91 |
| CK-192h-1-2 | 40209484 | 39062546 | 6.03 | 5.86 | 97.15 | 0.03 | 96.93 | 91.55 | 45.06 |
| CK-24h-1-1 | 40718166 | 39971544 | 6.11 | 6.00 | 98.17 | 0.03 | 97.03 | 91.75 | 45.98 |
| CK-24h-1-2 | 40935408 | 39932332 | 6.14 | 5.99 | 97.55 | 0.03 | 97.02 | 91.74 | 45.71 |
| CK-96h-1-1 | 47709226 | 46140318 | 7.16 | 6.92 | 96.71 | 0.03 | 97.37 | 92.63 | 45.24 |
| CK-96h-1-2 | 43519248 | 42820314 | 6.53 | 6.42 | 98.39 | 0.03 | 96.99 | 91.68 | 46.15 |
